# Health of the School Child

**Published:** 1923-12

**Authors:** 


### HEALTH OF THE SCHOOL CHILD.

### SIR GEORGE NEWMAN'S REPORT.

SIR GEORGE NEWMAN, Chief Medical Officer of the Board of Education, has issued his Report on " The Health of the School Child in 1922 " (His Majesty's Stationery Office. 1s. 6d.). In spite of financial restrictions which have arrested many developments proposed in recent legislation and the unparalleled conditions of unemployment there was no real reaction in 1922, "and the machinery which Local Education Authorities had laboriously and carefully built up in recent years," though denied expansion, was not severely damaged. The " most serious and wasteful defect in our national health service " is, he states, the lack of adequate supervision of the pre-school child.

### Infant Welfare.

It may be safely assumed that from 80 to 90 per cent. of children are born healthy and with the potentiality of leading normal and healthy lives, yet 35 to 40 per cent., when admitted to school, bear signs of physical defects :—

Whatever be the facts of parentage, the tendency of nature is to reassert the right of each new generation to the heritage of healthy birth. The object of the measures adopted for Infant Welfare is to maintain that endowment of good birth, and herein lies the importance of the work of the local Health Authorities, acting in conjunction with voluntary agencies, on behalf of the new-born child. This work increases from year to year, yet, comprehensive as it has become in recent years, the close supervision of the infant extends but little beyond the first year. There are, no doubt, adequate reasons to account for the limited supervision of the infant during pre-school life, such as the absence of compulsion on the parent to bring the child up to the centre, the difficulty in an increasing family of bringing up the elder babies together with the younger infants, who demand more immediate attention, and, lastly, the present financial position, which entails strict economy in regard to staff and remedial measures.

### More Co-Operation.

Sir George Newman thinks that more co-operation is required between the Infant and Child Welfare Centre, the Day Nursery and the Nursery School :—

The matters which require to be sifted and presented in each area include the age incidence and cause of crippling, infectious and other diseases of infancy ; the effect of neglect of these diseases on the general health of the child ; the means and institutions now existing for the prevention and amelioration of infant defects, and for securing true and effective nurture of the young child ; a considered scheme of child welfare before school days begin—a health scheme which shall be elastic enough to comprise all children of pre-school age in need of assistance or supervision.

### The Child of School Age.

Other urgent questions are the effective education of the dull or backward child, the importance of a comprehensive scheme for the treatment of dental defects, and the systematic organisation of the teaching of hygiene and physical training. The findings of medical inspection of children of school age show that definite malnutrition, heart disease, anæmia and deformity are present in about 2 per cent. of the children :—

Broadly, 100,000 children are suffering from each of these four conditions in the country as a whole. They are serious conditions in childhood and may prove disabling in after life. A larger burden of disease consists of defective vision, 20 per cent. ; ear, nose and throat disease, 14 per cent.; and a severe degree of dental disease, 34 per cent. Of the defective vision we may say that 10 per cent. of the children suffer from relatively serious defect.

### Treatment of Defectives.

With reference to the treatment of defective children we are still in a transition period :—

Owing to the skill of many experts, to the patience and devotion of many workers on After-Care Committees, and to the public spirit of the more enlightened Authorities, substantial progress is being made. What is needed is that all Education Authorities should recognise their responsibility in this matter and, co-operating together, do the best that is immediately practicable, pending firmer knowledge and better times as regards finance. I venture to make the following recommendations: Every School Medical Officer should ascertain the total number of mentally defective children in his area, and differentiate backwardness from specific defect, and such defect from idiocy, imbecility or delinquency. On the medical ascertainment the Authority should, as far as practicable, " clear " its cases, the definitely educable defective to the Special Schools, the definitely ineducable mental cases to the Local Control Authority.

### Results of Dental Inspection.

Dental sepsis is widespread among school children, although there is a very great and cumulative improvement over the last ten years :—

Between 1913 and 1919 the improvement amounted roughly to 5 per cent. more children leaving school with sound teeth ; in two years, from 1919 to 1921, another 5 per cent. was added ; and in the last year, 1922, still another 5 per cent., making 15 per cent. in all in nine years. The astonishing progress of the last three years is due to the fact that children are now leaving school who, nine years ago, first came under dental inspection and treatment. Last year it was regarded as a noteworthy achievement that some 8,000 boys and girls in London were leaving school with sound teeth who would not have done so had it not been for the preventive and curative measures introduced in connection with the school medical service. That this number would so soon be raised to 12,000 was scarcely then anticipated, and the result is, therefore, all the more inspiriting.

### Enlightening Public Opinion.

As preventive dental measures the Report suggests :—

A well-organised dental clinic is one of the best means of prevention and possesses also great educational value. The services of dental officers in giving instruction to parents and children should be placed upon a systematic basis by the Education Authority. In areas which are incompletely staffed, lectures on prevention should be given by approved lecturers, and where practicable illustrated by cinema films, lantern slides, charts or models.

### Secondary Schools.

Eight hundred and fifty secondary schools were examined last year at a cost of about £50,000 :—

The three defects most marked in Elementary School children (defective vision, dental decay and diseases of the nose and throat) are likewise the commonest among Secondary School children. Deformities are relatively high and suggest that physical training and games are neglected. Indeed, it has been found that physical training in many Secondary Schools is exceptionally bad and unsatisfactory.

The net expenditure on school medical service in 1922 was £1,354,140.

